# The Relationship between Intimate Partner Violence and Online Help-Seeking: A Moderated Mediation Model of Emotion Dysregulation and Perceived Anonymity

**DOI:** 10.3390/ijerph19148330

**Published:** 2022-07-07

**Authors:** Heng Xu, Jun Zeng, Zheng Cao, Huihui Hao

**Affiliations:** 1 School of Management, Henan University of Technology, Zhengzhou 450001, China; junzeng@stu.haut.edu.cn; 2 Graduate School of Management, Management and Science University, Shah Alam 40100, Malaysia; 012020073324@gsm.msu.edu.my; 3 Faculty of Agribusiness and Commerce, Lincoln University, Lincoln 7647, New Zealand; huihui.hao@lincolnuni.ac.nz

**Keywords:** intimate partner violence, willingness of online help-seeking, emotion dysregulation, perceived anonymity, social networking site

## Abstract

During the COVID-19 pandemic, lockdowns and isolation have limited the availability of face-to-face support services for victims of intimate partner violence (IPV). Despite the growing need for online help in supporting IPV victims, far less is known about the underlying mechanisms between IPV and online help-seeking. We studied the mediating role of emotion dysregulation (ED) and the moderating role of perceived anonymity (PA) on the internet to explain IPV victims’ willingness of online help-seeking (WOHS). Through a PROCESS analysis of the questionnaire data (*n* = 510, 318 female, 192 male, M_age_ = 22.41 years), the results demonstrate that: (1) ED has been linked with the experience of IPV, and IPV significantly induces ED. (2) When IPV victims realize the symptoms of ED, they have a strong willingness to seek external intervention to support themselves. ED mediates the relationship between IPV and online help-seeking. (3) For youth growing up in the era of social networking sites (SNS), personal privacy protection is an important factor when seeking online help. The anonymity of the internet has a positive effect on victims who experience IPV and ED, and it increases WOHS. This study introduces a new perspective on the psychological mechanism behind IPV victims’ help-seeking behaviors, and it suggests that the improvement of anonymity in online support can be an effective strategy for assisting IPV victims.

## 1. Introduction

Throughout the COVID-19 pandemic, intimate partner violence (IPV) has remained a serious and prevalent public health concern [1]. The rate of physical IPV in 2020 during the COVID-19 pandemic was 1.8-fold higher than in 2017–2019 [1]. Some serious negative health consequences result from IPV victimization, including emotion dysregulation (ED), post-traumatic stress disorder (PTSD), depression, anxiety, and sexual risk behavior [2,3,4]. IPV victims have been utilizing different technologies to seek help and support and to escape from abusive relationships, especially during the pandemic [5]. However, lockdowns, social distancing, and voluntary self-isolation reduce the options for social support [5].

Social media is crucial for people seeking help when experiencing IPV, and it is being used as a new way to seek help [6]. Social networking sites (SNS) have been integrated into young people’s daily lives, extending to their help-seeking behavior [7]. Social media has many advantages in the process of help-seeking, such as aiding the identification of abusive behaviors within a partnership, promoting awareness of available IPV resources, encouraging disclosure of IPV, and encouraging seeking assistance to leave a violent relationship [5]. In China, people are reluctant to make private family affairs public due to strong cultural norms about saving face. These oriental cultural norms have the potential to impede help-seeking preferences and behaviors [8]. Compared with western communities, individuals from Asia have more concern about “face”, feel greater discomfort in self-disclosure, and perceive fewer benefits of help-seeking [9]. Wong et al. [8] proposed that the anonymous nature of online help may be especially attractive to Asians with mental health concerns. Therefore, with the emergence of social media, IPV has gradually changed from the private sphere to the public sphere. The high freedom and openness of social media enable the public to break through the boundaries of time and space, understand IPV events, and express their personal views directly [6]. The anonymity and interactivity of the Internet help people break through social inhibitions in real society, and so they can express their real emotions truthfully or exaggeratedly.

In this context, research on the relationship between IPV and seeking help on social media began to emerge [5,10,11]. IPV victims often obtain informational resources and emotional encouragement through social media [5]. Fryc et al. [12] found that IPV often causes mental health problems in the victim, and mental health problems are a major cause of inducing people to seek help online [7,13]. In addition, the anonymity of SNS can help people overcome the obstacles of face-to-face service and increase their willingness to seek online help. Despite a recent surge of interest in this field, the literature is still sparse. Meanwhile, some gaps need to be filled in the existing literature. First, prior studies have focused heavily on the properties and sources of social support, but few studies have investigated the reasons why IPV victims are willing to seek online help from the perspective of social networks. Secondly, prior studies have shown that mental health symptoms prompt people to seek help, yet there is little research exploring how ED affects the willingness of online help-seeking (WOHS) from the perspective of the psychological mechanisms active in IPV victims. Furthermore, the relationship between IPV and ED has been unknown. Thirdly, as the main feature of social media, anonymity is beneficial to IPV victims in finding solace and social support in SNS. However, anonymity’s influences on WOHS have not been clearly understood. Therefore, it has become necessary to probe deeply into the mediating mechanisms and functions of anonymity and ED that play a crucial role for IPV victims‘ WOHS.

The present study aims to develop a moderated mediation model to explain the association between IPV and WOHS in young people. According to the aforementioned studies, we propose that ED mediates the association between IPV and WOHS, while perceived anonymity (PA) can moderate the mediating effect of ED.

## 2. Theoretical Background

### 2.1. IPV, Online Help-Seeking, and PA

IPV is defined as physical, sexual, or psychological harm committed by a current or former intimate partner or spouse. Seeking social support is a protective measure to cope with IPV and reduces the negative psychological and physical consequences of victims [5]. As the demand for formal services increases, online help-seeking, which can provide good support, is becoming increasingly common [11]. Chu [5] proposed that SNS could be an appropriate space for women who are victims of domestic abuse to seek social support, and emotional support requests were the most prevalent type, followed by informational support requests. Online support can overcome many barriers associated with face-to-face support and can provide greater control over the help-seeking process [11]. Moreover, online support can offer additional advantages such as increased anonymity, privacy, and flexibility.

Anonymity means a lack of identification of one’s real identity [14]. Hite et al. [15] defined PA as the extent to which individuals perceive that their personal identity is unknown to others or that they are unidentifiable as an individual. In the social identity model of deindividuation effects (SIDE), the effects of online anonymity are reflected in personalization, misconduct, and false information, which are related to the dark side of cyberspace [16]. However, anonymity in cyber-based communication may not necessarily lead to antisocial behavior [17]. In some scenarios, anonymity enhances social processes associated with group identity in online communication and can also play a positive role in information exchange [18,19].

The perception of anonymity refers to the indiscernibility of the identity of the user, or that one cannot be tracked in cyberspace [20]. Therefore, PA has a positive effect on self-disclosure [19]. People may prefer to share content and information online anonymously without worrying about the reactions of others [21]. In the field of online health services, anonymity facilitates engagement and disclosure via formal pathways [22]. In addition, young people who perceive that they are in an anonymous environment are more likely to disclose themselves and seek online health services.

Table 1 summarizes the related literature on the relationship between IPV and online help-seeking. The focuses of scholars in the field of IPV include the difference between online help and face-to-face help [11], associations and implications for self-help and online interventions [10], examining the nature and content of messages about IPV [5], etc. In addition, when facing mental health disorders, young key populations require appropriate cultural and developmental interventions [23]. Troubled individuals choose to seek help from online support groups rather than face-to-face options because of the benefits of online help, including the elimination special barriers, flexibility, and the option of maintaining anonymity, which provides emotional security, as well as a preference for written communication [24]. These characteristics increase the proliferation of online support groups [24]. However, in China, a majority of young people are reluctant to seek help from mental health services because of the lack of anonymity, feeling shame, and a desire to save face [25]. In fact, offering online help, such as intervening via chat, could be a solution to remove these barriers and reach young people [22]. Online interventions in this space have shown a great deal of theoretical promise [11].

### 2.2. IPV and ED

Research and theory suggest that ED is closely correlated with experiencing IPV [12]. IPV is associated with several poor health outcomes, including ED [12,27]. ED is characterized by maladaptive responses to emotions, including: (1) a lack of awareness, understanding, and acceptance of emotions; (2) the inability to control behavior when experiencing emotional distress; (3) a lack of access to situationally appropriate strategies for modulating the duration and intensity of emotional responses in order to meet individual goals and situational demands; and (4) an unwillingness to experience emotional distress as part of pursuing meaningful activities in life [29].

ED is one symptom that may be especially relevant to victims with a history of IPV. Preliminary research has linked emotion regulation difficulties to ED in both youth and adult populations [30]. Lagdon et al. [31] suggested that women who had experienced IPV had more frequent and intense emotional distress than those who had not experienced IPV because IPV made it difficult for women to effectively modulate their emotions, including depression and anxiety. Male victims also face similar difficulties. Audet et al. [26] found that men who seek help for IPV-related difficulties often have high levels of psychological distress and emotional disorders. Therefore, IPV has a negative impact on emotional regulation (ER), which results in ED symptoms.

In order to reduce emotional distress, IPV victims rely on social support [28]. For young people, in particular, Rickwood et al. [13] proposed a conceptual model of help-seeking in which the behavior of advocating seeking help begins with being aware of and expressing mental health problems, and then recognizing the availability of support services, and, finally, generating a willingness to seek help. When IPV victims become aware of their own needs and desires, self-awareness enable them to make the necessary changes and to overcome barriers during the recovery process [32]. In addition, Sideridis et al. [33] proposed that emotion is a predictor of an individual’s tendency to seek help. Identifying ED symptoms can trigger individual coping strategies and alter one’s willingness to seek help. ED could be one factor that leads to WOHS; however, the function and mechanism that ED plays in the process of an IPV victim seeking help remains unknown.

In summary, the existing literature has studied the advantage of online help and the emotional consequences coming from IPV; however, there is a lack of literature on the mediating mechanism behind the willingness of IPV victims to seek online help.

## 3. Hypotheses Development

**Figure 1:**
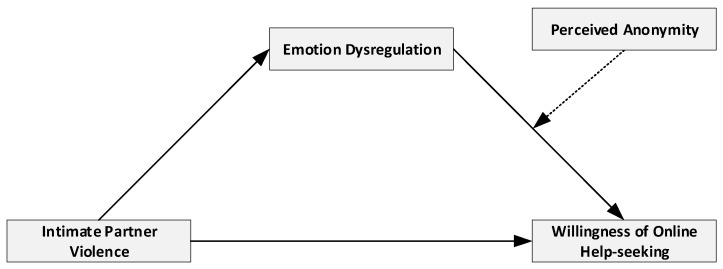
The proposed moderated-mediation model of the relationship between IPV and online help-seeking.

Figure 1 shows a conceptual model depicting the relationships between IPV, ED, PA, and WOHS. This proposed moderated-meditation model explains the mediator role of ED between the association of IPV and WOHS, and the moderator role of PA between the association of ED and the level of WOHS in young people.

### 3.1. The Relationship between IPV and WOHS

Some empirical research has demonstrated a strong association between IPV and the decision to seek help [34,35,36,37]. In general, help-seeking is defined as the process of locating and utilizing either formal (e.g., police, courts, or community advocacy) or informal (e.g., family or friends) resources for victimization support. Both informal and formal resources may mitigate the negative consequences associated with IPV [34]. In addition, IPV victims who experience controlling behaviors may be more likely to disclose and seek help [34]. Women of any age who experience IPV tend to seek help from informal networks, especially young women [37]. Accordingly, this study formulates the following hypothesis:

**Hypothesis** **1.** *There is a positive relationship between IPV and WOHS.*

### 3.2. The Mediating Role of ED

Muñoz-Rivas et al. [27] demonstrated that there is a general difficulty in regulating emotions among victims who have experienced IPV. According to the general model of ED by Fryc et al. [12], ED is one factor that may be especially relevant among survivors with a history of IPV. If women who have experienced IPV also have experienced ED, both psychotherapy and neurological consultation will be required for health [12]. Therefore, ED is a result of strain and predisposes one to help-seeking behavior [38]. Nevertheless, it is unknown whether ED can mediate the relationship between IPV and WOHS. Therefore, the following hypothesis is formulated:

**Hypothesis** **2.** *ED mediates the relationship between IPV and WOHS.*

### 3.3. The Moderating Role of PA

The anonymity associated with the online environment means it may be seen to offer greater levels of privacy and increased security, which may encourage self-disclosure [20]. The perception of anonymity refers to the indiscernibility of the identity of the user, or that one cannot be tracked in cyberspace [39]. PA is a well-known concept, and the studies that focus on PA indicate that it is strongly associated with online help-seeking and ED.

When young people have emotional disorders due to IPV, they might experience negative emotions, which in turn may exacerbate emotional regulation difficulties, leading to a need for external support and intervention. Wong et al. [9] have demonstrated a strong relationship between mental health and help-seeking preferences. The perceived benefits of online compared to face-to-face support include anonymity, ease of access, immediacy, and the opportunity to connect with those who have had similar experiences [7]. Moreover, these benefits associated with online help seem to eliminate the common help-seeking barriers to traditional sources of help, such as stigma and embarrassment, as well as concerns about confidentiality [9]. Therefore, this study formulates the following hypothesis:

**Hypothesis** **3.** *PA moderates the relationship between ED and WOHS.*

## 4. Methodology

The above hypothesis is proposed to determine the underlying mechanism of WOHS from that of PA and ED. This section studies that relationship through a quantitative survey. Compared with experimental methods, a questionnaire survey can help explain a phenomenon in more depth [15]. Following the recommendations of Suveg et al. [30], we first formulated a research model using insights gained from the literature review. This was immediately followed by a survey-based quantitative study to evaluate the research model.

### 4.1. Ethical Considerations

The ethics committee of the researcher’s university approved this project and the study, including the content of the questionnaire. We obtained electronic informed consent from the participants. The participants’ answers are completely anonymous to ensure confidentiality in the handling of the information. In addition, participants read a debriefing which explained the goals of the study, and they also had the opportunity to request an additional oral debriefing. We provided the email address at the beginning and end of this questionnaire and offered a free videoconferencing meeting with a psychologist that specialized in the field of IPV. None of the participants requested this assistance.

### 4.2. Procedure and Participants

To ensure the reliability and validity of the survey results, we distributed an online questionnaire within six social network groups in the researchers’ university before entering the formal distribution of questionnaire. Among the 302 responses, 11 questionnaires were invalid because the response time was less than three minutes. The statistical results of the pre-survey showed that the KMO value was 0.718 and Bartlett’s test of sphericity was significant (*p* < 0.001). A total of four factors were extracted and the variance contribution rate was 63.402%, which indicated that the variables had high measurement validity. In addition, the Cronbach’s α values of each variable were greater than 0.7, which indicated that the scale had internal consistency and could be used in formal questionnaires. The relevant data of the formal investigation are presented in Section 4.3.

The questionnaire was distributed by a university lecturer with a psychological counseling certificate, and it used a hyperlink via different social networking approaches (e.g., QQ group and WeChat group). The Wenjuanxing platform was the platform used for data collection for this online survey. The online survey included several scales (see the measures section below for details), demographic information, and electronic informed consent. The research purpose, eligibility for participation, and rights of withdrawal were clearly presented at the top of the first page of the Wenjuanxing forms. After participants accepted the informed consent, they could continue the survey.

The inclusion criteria were: (1) being a current university student, (2) having the ability to understand the Chinese questionnaire and the meaning of the questions, and (3) having experienced IPV in the past year. The questionnaires were carried out between January 2022 and March 2022. Of the total 569 participants who completed the questionnaire, 510 (female: 62.4%, male: 37.6%, average age: 22.41 years) valid questionnaires were collected after excluding the invalid questionnaires, such as a pattern of irregular responses and blank questionnaires.

### 4.3. Measures of Variables

(1) IPV

The Revised Conflict Tactics Scales (CTS2) was proposed by Straus and colleagues [40] and translated and revised into the Chinese version by Li et al. [41]. Psychological aggression included 10 questions, physical assault included 11 questions, sexual coercion included 8 questions and injury included 7 questions, such as “Has your partner ever insulted or sworn at you?” The current study utilized the section that included the IPV items. Meanwhile, referring to the pre-survey results and the advice of a psychological consultant, we added cold violence and self-harm in dating violence into the content of questionnaire [42,43]. A 5-point rating scale was used in this questionnaire to rate items (1 = 1–2 times in the past year, 2 = 3–10 times in the past year, 3 = 11–20 times in the past year, 4 = more than 20 times, 5 = not in the past year, but it did happen before, and 0 = this has never happened). In this study, the Cronbach’s α was 0.94, AVE was 0.628, and CR was 0.976.

(2) ED

The Difficulties in Emotion Regulation Scale compiled by Bjureberg [44] is a 16-item self-report measure of difficulties regulating negative emotions across five domains: nonacceptance of negative emotions, difficulties engaging in goal-directed behavior when distressed, difficulties controlling impulsive behaviors when distressed, limited access to effective emotion regulation strategies for negative emotions, and lack of emotional clarity. This scale was used by Simpson et al. [29] to measure the role of ED in the relation of IPV to PTS among women who experienced IPV. The questionnaire consists of sixteen items, such as “When I am upset, I become out of control”. Each item was rated on a 5-point scale, ranging from 1 (almost never) to 5 (almost always). Higher scores meant greater negative emotions. The validity of the scale has been demonstrated to be good. In this study, the Cronbach’s α was 0.97, AVE was 0.641, and CR was 0.964.

(3) PA

PA was measured using a scale initially developed by Hite et al. [15], which was used to validate the influence of PA on information-sharing behavior [21]. The scale consists of five items, such as “Those who read my posts, pictures or videos can guess my identity”. The items are rated on a 5-point scale ranging from strongly disagree to strongly agree. The total score of the PA is obtained by summing the ratings for each item, with higher scores indicating a higher PA quality. In this study, the Cronbach’s α was 0.86, AVE was 0.675, and CR was 0.858.

(4) WOHS

The General Help-Seeking Questionnaire (GHSQ) was measured using a scale initially developed by Deane et al. [45], to which additional help sources were added by Seward and Harris [46], such as online MHP (e.g., psychologist/psychiatrist), online support sites (run by a professional organization), social networks (e.g., Facebook or Twitter), and anonymous online forums. This scale was translated and revised into the Chinese version by Liu et al. [47]. The items are rated on a 5-point scale ranging from extremely unlikely to extremely likely. In this study, the Cronbach’s α was 0.90, AVE was 0.760, and CR was 0.926.

### 4.4. Analytical Method

This study used the SPSS statistical software (IBM, Armonk, NY, USA) to analyze the descriptive statistics and reliability of the measures. Furthermore, the Hayes PROCESS macro (Model 14) was used to test the hypotheses in this study. PROCESS performs ordinary least squares regression to estimate the moderated indirect effect. In the moderated mediation analysis, all of the quantitative variables were centralized [48], and the 95% percentile bootstrap confidence interval (CI) with 5000 bootstrapping samples was calculated. Confidence intervals that did not contain zero indicated significant effects. The index of moderated mediation and its 95% CI, as estimated by PROCESS, were used to determine and quantify the statistical significance of the moderated mediation effect [49]. The PROCESS macro has been widely used in the psychology field [27].

## 5. Results

### 5.1. Demographic Characteristics and Correlations among the Study Variables

Of the 510 valid participants, 62.4% (*n* = 318) were female, 37.6% (*n* = 192) were male, and the mean age was 22.41 years (SD = 0.75). Over half of participants (60.5%) had an existing relationship for more than a year. Most of the participants’ partners (91.6%) had a college education. A minority of the young adults (4.1%) never used SNS. From January 2021 to March 2022, more than a large majority of the participants (72.4%) had experienced psychological violence (spited: 65.3%, cold violence: 62.0%); almost half (40.4%) had experienced physical violence (slapped: 35.5%, pushed: 32.9%); and 31.0% had experienced sexual violence (forced without a condom 27.6%, forced sex: 27.8%). Specifically, 9.2% of the participants experienced IPV more than 10 times. 

As shown in Table 2, the mean scores of IPV and ED were 0.95 (SD = 1.21) and 2.37 (SD = 1.06), respectively. WOHS had a mean score of 3.12 (SD = 1.37). For each variable, skewness ranged from −0.08 to 1.06 and kurtosis ranged from −1.38 to −0.38. Based on reference values of an absolute skewness value of ≤2 or an absolute kurtosis of ≤4, the assumptions of normality for the variables were met. IPV was positively correlated with WOHS (r = 0.450, *p* < 0.01). ED was positively correlated with IPV (r = 0.503, *p* < 0.01) and WOHS (r = 0.318, *p* < 0.01).

### 5.2. Tests for the Moderated Mediation Model

**Figure 2:**
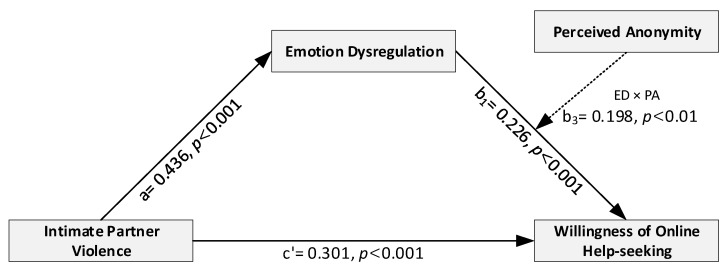
The coefficient estimates and statistical significance of the moderated-mediation model.

Model 14 of the PROCESS macro was used to test the study hypothesis, that is, to examine the relationship between IPV and WOHS and the mediating role of ED and the moderating role of PA. In all analyses, we controlled for covariates, including age, gender, partner’s education, and frequency of social media use. Figure 2 presents the regression coefficients for each path in the moderated mediation model. As shown in Table 3, the direct effect of IPV on WOHS was significant (path c’: β = 0.301, *p* < 0.001, 95% CI 0.170, 0.431). In the mediation analysis, IPV positively predicted ED (path a: β = 0.436, *p* < 0.001, 95% CI 0.352, 0.499), and ED positively predicted WOHS (path b_1_: β = 0.226, *p* < 0.001, 95% CI 0.106, 0.346). The findings support Hypothesis 2. Next, the moderated mediation showed a significant interaction effect between ED and WOHS (path b_3_: β = 0.198, *p <* 0.01, 95% CI 0.069, 0.326). In summary, these results supported the positive, indirect effect of IPV on WOHS through the positive mediating effect of ED. 

**Figure 3:**
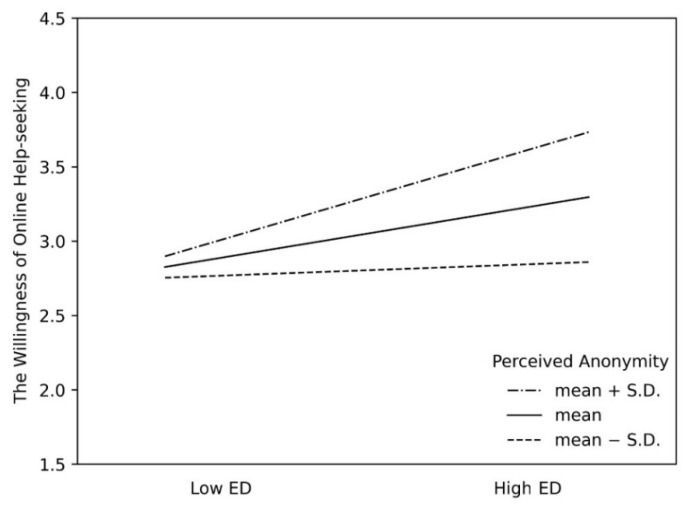
The moderating influence of PA on the association between ED and WOHS.

To better understand the moderating effect of PA, the bootstrap indirect effects were estimated for the mediating effect of the severity of ED at three different levels of PA (mean plus SD, mean, and mean minus SD). More detailed information is shown in Figure 3 and Table 4. The 95% CI shows that when the PA level is high, ED has a strong positive impact on WOHS. When the PA level is lower, ED has a positive impact on WOHS. However, this effect was lower than that of the high-perceived-anonymity group, which is illustrated in Figure 3. To sum up, the moderating effect of PA was confirmed, as the mediating effect of ED in the association between IPV and WOHS was stronger at higher levels of online anonymity, thus indicating the protective effect of PA.

## 6. Discussion

Quantitative validation of the proposed model confirmed that ED could have mediating effects on IPV victims in generating WOHS. This mediating mechanism is the process by which IPV victims become aware of ED symptoms, and this indirect relationship between ED and WOHS is moderated by PA.

First, in the era of the Internet, people have more online resources for help. Almost 70% of young people report that they have previously accessed some form of online support for personal or emotional problems [50]. This includes informal information via social media and formal counselling services. The results show that IPV is positively associated with WOHS (Hypothesis 1). This is in line with the findings of Chu et al. [5]. Moreover, SNS can be an important channel for women who are victims of domestic abuse to disclose their situation, seek social support, obtain valuable information, and receive emotional encouragement [5]. Seeking help can help reduce mental health risks such as depression and anxiety, improve the skills of conflict negotiation and management, and protect individuals from further harm by violence.

Hines et al. [51] pointed out that mental health problems are major concerns for male IPV victims. In our questionnaire, one third of the participants were men. The results showed that 42.7% of male victims had suffered from emotional disorders for a long time in the past, and 47.4% of male victims would be willing to seek online help. This verifies that both men and women can be partner abusers [52], and indicates that male IPV victims also have a need to seek help [51]. However, studies assessing the health concerns related to IPV victimization tend to focus on women, rather than men [53]. Therefore, research on online help-seeking for male victims needs to be explored further.

Second, the findings revealed that ED mediates the relationship between IPV and WOHS. The previous study demonstrated that IPV could cause ED [21]. Our findings further demonstrate that ED could stimulate IPV victims to have WOHS (Hypothesis 2). The negative emotions coming from IPV tend to deplete the victim’s energy and reduce their ability to adaptively cope with situational challenges [54]. The inability to manage emotionally arousing experiences potentially influences anxiety symptoms [30]. A high proportion of participants expressed a preference for online resources, which may have been influenced by the stigma associated with mental health problems [25]. Tarzia et al. [11] proved that a majority of the participating women preferred accessing Internet resources for their mental health problems. Therefore, young people with high levels depression or anxiety prefer seeking mental health information on the Internet [55]. In summary, when IPV victims feel overwhelming, intensely negative emotions and cannot regulate their emotions, they have a strong willingness to obtain an external intervention to defend themselves.

Especially in China, people are influenced by the traditional cultural norms of face-saving and preserving family honor, which leads to the avoidance of topics such as domestic violence and IPV. The public’s awareness of IPV is deficient, and many victims have a high tolerance for it. In this cultural context, when IPV victims are willing to ask for help, seeking online resources becomes the first choice.

Finally, In the study completed by Horgan et al. [56], 22.3% of participants preferred online mental health services to face-to-face services because they are anonymous, private, and confidential. Anonymity can reduce stigma and embarrassment when seeking help. As a result, ensuring that websites maintain young people’s confidentiality and anonymity appears to be critical for increasing the usage of online services [57]. Our results go one step further and demonstrate that increasing the anonymity of online services can increase IPV victims’ willingness to seek help under the moderation of PA (Hypothesis 3). It shows that anonymity is the facilitator of online help-seeking compared to face-to-face services. A higher level of anonymity when using SNS has a positive effect on victims who have experienced IPV and ED, and it increases the possibility of WOHS.

## 7. Conclusions

Throughout the COVID-19 pandemic, there has been an increasing demand for online help-seeking for IPV victims. This research provides valuable insights to explain the psychological mechanisms underlying the relationship between IPV and WOHS in SNS. (1) We examined the mediating effect of ED. IPV can significantly induce ED symptoms in victims. When IPV victims perceive their own ED symptoms, they will have a strong willingness to seek online help for the purpose of psychological adjustment and self-defense. (2) We examined the moderating effect of PA. An IPV victim’s perception of social network anonymity can significantly influence WOHS in IPV victims who have symptoms of ED. The higher the degree of PA, the greater the willingness to seek online help.

### 7.1. Theoretical Contributions

Firstly, focusing on online help-seeking, this study provides a new perspective. The previous research mainly focused on offline social intervention [23,58]. However, in the context of the COVID-19 pandemic, for IPV victims, online help is an important public health service, and the Internet provides more convenient support sources for people seeking help. This study further validates the conclusion of Aguilera-Jiménez et al. [52] by drawing on a larger sample of men. Consequently, this study enriches theories in the field of social help.

Secondly, this study identifies a new psychological mechanism underlying an IPV victim’s WOHS. Although previous studies have shown that IPV causes PTSD [27], we further found that IPV can also induce ED symptoms in victims. When victims feel ED, they will have WOHS. Therefore, ED plays a mediating role in the relationship between IPV and WOHS.

Finally, this study pushes forward the moderating role of PA on SNS in the willingness of IPV victims to seek online help. Existing research suggests that most IPV victims are reluctant to seek outside assistance or social support [34,59]. However, when IPV victims have a high degree of PA in social networks, they realize that their privacy is fully protected, resulting in a strong willingness to seek help online. This finding complements previous literature studies [55].

### 7.2. Implications for Practice

The findings of this study add to the knowledge base on the use of IPV interventions in the setting of SNS. Online help services act as a significant way in which IPV victims can obtain informational resources and emotional encouragement. Making people feel private and secure can address their concerns and increase WOHS. There is a definite need for more victim-centered, particularly male-friendly, practice approaches to ensure that a victim’s negative effects are lessened, while simultaneously empowering them [60]. This study suggests that online service organizations should extend professional training and service provision while respecting and valuing privacy, as well as provide anonymous services and secure access for IPV victims.

### 7.3. Limitations and Future Research Directions

The present study has certain limitations that open avenues for future research. First, it is more convenient to collect data using a questionnaire survey, but when there are many items on the scale, there may be some deviations in the measurement of participants’ psychology. In future studies, we will use experimental methods to further verify the reliability of the model and the robustness of the conclusions. Second, the existing research shows that the perception of social support has a positive effect on seeking help, especially for informational resources and emotional encouragement that IPV victims can obtain from the online community. In future research, online social support can be considered as a moderating factor in order to better understand the underlying psychological mechanisms of IPV victims seeking online help.

## Figures and Tables

**Table 1:** Summary of the related literature.

Author (Year)	Objective/Context	Methodology	Results/Findings
Tarzia et al. (2018) [11]	The gap between online support and face-to-face support for women who had experienced IPV	RCTs	Many key elements of face-to-face support for IPV, such as awareness-raising and lessening isolation, can be provided well via the Internet, which can offer additional advantages in some areas, such as increased anonymity, privacy, and flexibility of access, and provide safety mechanisms.
Roddy et al. (2018) [10]	Associations and implications for self-help and online interventions	Hierarchical linear modeling	Web-based interventions may be an effective (and easily accessible) intervention for relationship distress for couples with low-intensity IPV.
Chu et al. (2021) [5]	Examine the nature and content of messages presented in a Chinese online community about IPV	Quantitative content analysis and automatic content analysis	Most of the messages provided informational support in terms of personal experience, explanations, and strategies for coping with IPV, as well as emotional support regarding wishes, encouragement, and empathy for the victims.
Audet et al. (2022) [26]	Examine whether psychological distress symptoms are indirectly related to the perpetration of IPV through affect dysregulation	Questionnaire survey	Symptoms of anxiety were directly related to lower physical assault perpetration and indirectly related to higher physical assault and coercive control perpetration through higher affect dysregulation.
Muñoz-Rivas et al. (2021) [27]	Determine the variability of ED among women with different types of IPV revictimization and post-traumatic stress	Questionnaire survey and cluster analysis	The Emotional Overwhelm group was characterized by a general dysregulation of emotional experiences and a greater intensity of post-traumatic stress symptoms. ED is a critical pathway to the decrease of health among IPV victims.
Fryc et al. (2022) [12]	The potential influence of presumed head and neck injuries from IPV on ED	Questionnaire survey	There is an association between presumed head and neck injuries from IPV and ED, underscoring the potential need for considering both neurological and psychological factors in the assessment and treatment of ED in this population.
Schwank et al. (2020) [28]	Assess Shanghai women’s care-seeking behavior for mental health disorders	Questionnaire survey	A total of 82.2 percent seek online support. Shanghai women avoid seeking professional help for mental health issues. Friends, spouses, and online resources are the preferred venues.

**Table 2:** Distribution, normality estimates, and correlation matrix for all variables.

Variables	1	2	3	4
1. IPV	1	-	-	-
2. ED	0.503 **	1	-	-
3. WOHS	0.450 **	0.318 **	1	-
4. PA	0.586 **	0.296 **	0.385 **	1
Mean (SD)	0.95 (1.21)	2.37 (1.06)	3.12 (1.37)	3.37 (0.88)
Skewness	1.06	0.33	−0.08	0.07
Kurtosis	−0.38	−0.79	−1.38	−0.68

Notes: ** *p* < 0.01; SD: standard deviation.

**Table 3:** Ordinary least squares regression results for moderated effect.

Outcome	Predictors	Path	β	SE	*p*	LLCI	ULCI
ED	IPV	a	0.436	0.037	<0.001	0.352	0.499
	R^2^ = 0.26, F = 35.05, *p* < 0.01						
WOHS	IPV	c’	0.301	0.067	<0.001	0.170	0.431
	ED	b_1_	0.226	0.061	<0.001	0.106	0.346
	PA	b_2_	0.297	0.074	<0.001	0.151	0.442
	ED × PA	b_3_	0.198	0.065	<0.01	0.069	0.326
	R^2^ = 0.27, F = 22.73, *p* < 0.01						

Notes: LLCI: lower limit of 95% confidence interval; ULCI: upper limit of 95% confidence interval; β: regression coefficient; SE: standard error.

**Table 4:** Moderated effect divided into three levels of perceived anonymity.

Perceived Anonymity	Indirect Effect	Bootstrap SE	95% of CI ^1^
Mean − SD	0.0222	0.0386	(−0.0595, 0.0951)
Mean	0.0962	0.0347	(0.0277, 0.1644)
Mean + SD	0.1701	0.0532	(0.0709, 0.2785)

Notes: ^1^—percentile bootstrap confidence interval.

## Data Availability

The data presented in this study are available on request from the corresponding author.
